# Evaluation of Phytochemical Content and In Vitro Antioxidant Activities of *Pistacia lentiscus* L. Leaves Extracts, a Medicinal Plant From the Beni-Snassen Forest (Eastern Region of Morocco)

**DOI:** 10.1155/2024/9999175

**Published:** 2024-10-24

**Authors:** Mourad Bendada, Abderrahmane Hadini, Ouahid El Asri, Youness Taarabt, Abderrahmane Nazih, Karim Andich, Khalid El Bekkaye, Khalid Chaabane

**Affiliations:** ^1^Laboratory of Agricultural Production Improvement, Biotechnology and Environment, Faculty of Sciences, Mohammed First University, Box 717, Oujda 60000, Morocco; ^2^Laboratory of Microbial Biotechnology and Vegetal Protection, Faculty of Sciences, Ibn Zohr University, Box 8106, Agadir 80000, Morocco; ^3^Laboratory of Soil Sciences, The National Institute of Agronomic Research, Box 428, Oujda 60000, Morocco

## Abstract

A medicinal plant from the Beni-Snassen Forest in the eastern region of Morocco has been studied. This scientific research was carried out to measure the content of essential phytochemical constituents and their antioxidant capacities from the hydromethanolic extract of leaves of *Pistacia lentiscus* L. located on sites at varying levels of altitudes. Our results have shown that at the lowest altitude, there was a height significant (*p* ≤ 0.05) in the content of flavonoids and polyphenols. On the other hand, the ascorbic acid, chlorophylls, and tannins had a higher content concentration on sites with high altitudes. Analysis correlation shows a hight correlation between the DPPH and the polyphenols content. A correlation between the total antioxidant activity and flavonoid content was found to have many similarities. The results indicated that *P. lentiscus* L. leaves have significant sources of chemical compounds that might be employed for various purposes.

## 1. Introduction

Morocco kingdom is a country known for its originality in the Mediterranean region due to its extreme ecological diversity in terms of vegetation, bioclimate, fauna, and morphology. The Moroccan flora is rich and diverse. This diversity is at the origin of the richness of the landscapes, geographical position, natural environments of high quality, and climatic factors. The Moroccan Forest is the founding element of the two thirds of the ecological wealth of the country; it shelters two thirds of the plants. Paromatic and medicinal plants) are an essential part of this biodiversity. In this context, we cite *Pistacia lentiscus* L., a medicinal plant belonging to the family Anacardiaceae, commonly referred to as lentisk, distributed in many areas in the Mediterranean ecosystems [1, 2], characterized by irradiations and high temperatures and the scarcity of water and necessary nutrients, due to prolonged drought [3, 4]. Recent studies have shown that the leaves of this medicinal plant contain several bioactive molecules, such as polyphenolic compounds [5, 6], including gallotannins and flavonoids [7–10]. These secondary metabolites possess anticancer, anti-inflammatory [8, 11], antiproliferative [12], antioxidant [13], antimicrobial [14], skincare, and antiaging properties [15, 16]. Indeed, the quality of these secondary metabolites is influenced by several factors, including the technological extraction process involved in their extraction, the leaf age, seasonality, and the harvest moment [7, 17–21]. *Pistacia lentiscus* leaves have been utilized for a long tradition in folk medicine. They have been employed to treat various diseases, such as hypertension, stomach aches, and kidney stones [22–25]. In addition, leaf extracts have been shown to have antiulcer, anti-inflammation, cytoprotecting, acetylcholinesterase inhibitory, and anticancer activity [22, 26–28]. In previous studies, the impact of environmental factors on the chemical compounds of the leaves of *Pistacia lentiscus* is poorly studied. Therefore, this research study aimed to evaluate the effect of the environment (altitude factory) on phytochemical constituents and in vitro explored the antioxidant activity of leaf extract of *Pistacia lentiscus* L. from the eastern region of Morocco at different levels of altitudes (m).

## 2. Materials and Methods

### 2.1. Collection of Plant Material and Processing

Leaves from the medicinal plant *Pistacia lentiscus* L. were collected from various sites in the Beni-Snassen Forest, located in the province of Berkane (Eastern Region of Morocco) in July 2022. The plant was botanically identified as *Pistacia lentiscus* L. with the support of the National Institute of Agronomic Research, Morocco. Samples were taken from three randomly selected individuals at six different altitude levels. The altitudes of the collection sites were as follows: S1 = 1100 m, S2 = 1010 m, S3 = 800 m, S4 = 670 m, S5 = 500 m, and S6 = 390 m. After collection, the samples were cleaned to remove any foreign matter, as well as any damaged or pest-infected leaves. The leaves were then shade-dried for three weeks, milled, and finally stored in polyethylene bags at −20°C until further use.

### 2.2. Preparation of Hydromethanolic Extracts

In the current study, based on the demonstrated effectiveness of methanol as an extraction solvent in our preliminary studies, where hydromethanolic extracts yielded higher amounts of bioactive compounds compared to other solvents, a hydromethanolic solution was used [29, 30]. Extraction was performed by maceration with an agitator at 25°C of fine powder in 80% methanol (*v/v*) for 48 h. After maceration, the obtained extract was filtered. Finally, a methanol solution at a concentration of 50 mg/mL (*v/v*, 80%) was maintained at −20°C until future use.

### 2.3. Phytochemical Indices Estimation

#### 2.3.1. Determination of Phenolics and Flavonoids

##### 2.3.1.1. Total Phenolic Content (TPC)

The measurement of the TPC in the leaves extract of *Pistacia lentiscus* L. was performed following the method of Folin–Ciocalteu described by Velioglu et al. [31]. In brief, 500 μL of the diluted extract from each sample was added to 250 μL of Folin–Ciocalteu reagent and 3.75 mL of distilled water. After a few minutes, Na_2_CO_3_ solution (0.5 mL, 20%) was added to the resultant solution after 5 min; then, the mixture was stored in the dark (for 30 min). Using the spectrophotometer, the absorbance of the solution obtained was measured at 760 nm. TPC was expressed as mg GAE/100 g DM.

##### 2.3.1.2. Total Flavonoids Content (TFC)

TFC was quantified using the aluminum chloride colorimetric method, described by Koolen et al. [32]. In brief, a volume of 500 μL of the methanolic solution of 2% AlCl_3_ (aluminum chloride, *p/v*) was mixed with a volume of 500 μL of the diluted extract. Incubation was carried out for 40 min at 25°C. After that, the absorbance is then measured at 430 nm using a spectrophotometer. Results obtained were expressed as mg RE/100 g DM.

#### 2.3.2. Content of Condensed Tannins (CCTs)

The vanillin method described by Julkunen [33] was used to measure CCT in extracts. Each extract (50 μL) was added to 1500 μL of the vanillin ethanol solution (4%). Concentrated HCl (750 μL) was added to the resultant solution. The absorbance readings of the sample were recorded at 550 nm post 20 min incubation at 25°C. The concentrations of condensed tannin (CT) were expressed as μg CE/100 g DM.

#### 2.3.3. Determination of Ascorbic Acid (AscA) Contents

AscA determination was performed according to the modified method of Mau et al. [34]. Both the dried leaves (1 g) and oxalic acid (10 mL, 1%) were mixed and stirred for 15 min. After that, we filtered the result and mixed 1 mL of 2,6-dichlorophenolindophenol (5 mM DCPIP) with 3 mL of the filtrate. After 15 s, the absorbance was measured at 515 nm. The content of AscA was expressed in mg AscAE/100 g DM.

#### 2.3.4. Measurement of Photosynthetic Pigment Contents

To determine the total chlorophyll, chlorophyll a, and chlorophyll b content of the lentisk leaves, we used 20 mL of acetone (80%) to grind about 0.2 g of fresh leaf sample. The mixture was incubated and placed in the dark at 25°C for 48 h [35]. Absorbance (Abs) was measured at 663, 645, and 652 nm using a spectrophotometer. The readings were expressed as μg/mL. The photosynthetic pigment contents were calculated using the following formulas:

(1) $$\begin{array}{c} \text{Chlorophyll }a=12.21\times {\text{Abs}}_{663}-2.81\times {\text{Abs}}_{645}, \end{array}$$

(2) $$\begin{array}{c} \text{Chlorophyll }b=20.13\times {\text{Abs}}_{645}-5.03\times {\text{Abs}}_{663}, \end{array}$$

(3) $$\begin{array}{c} \text{Total chlorophyll}=\text{chlorophyll }a+\text{chlorophyll }b. \end{array}$$

### 2.4. In Vitro Antioxidant Assays

#### 2.4.1. 1,1diphenyl-2-picrylhydrazyl (DPPH) Radical Scavenging Assay

To determine DPPH radical scavenging assay, we prepared 1 mg/mL of the initial extract (50 mg/mL). DPPH assay was performed according to the modified method of Braca et al. [36]. In brief, in this assay, 0.1 mL of the extract was mixed with 2.9 mL of DPPH, 6.10^−5^, in methanol, and incubated at 25°C for 30 min. The readings of Abs were taken at 517 nm. As a control, we used distilled water. The inhibition activity of DPPH expressed in percentage is calculated as follows:

(4) $$\begin{array}{c} \left(\%\right)\text{ Inhibition activity}=\frac{\left(\text{Abs control}-\text{Abs extract}\right)}{\text{Abs control}}\times 100, \end{array}$$

where Abs control was the absorption of the DPPH and Abs extract was the absorption of the plant extracts.

#### 2.4.2. Phosphomolybdenum-Based Total Antioxidant Activity Determination

To determine the total antioxidant activity of *Pistacia lentiscus* L. leaves extract, we use the method described by Umamaheswari and Chatterjee [37]. First, in a water bath, maintained at 95°C, we incubated 100 μL of the extract (1 mg/mL) with 1 mL of the phosphomolybdate reagent solution for 90 min. The readings of Abs were taken at 695 nm. The results are presented in mg ETOC/100 g DM. All experiments were repeated thrice.

### 2.5. Statistical Analysis

All statistical analysis of our results was performed by IBM SPSS Statistics 21. Data are presented as the mean values ± SD. To analyze the variance of our results obtained depending on the altitude (m) as an environmental factor, we used one-way variance analysis. To classify the means, the Student Newman–Keuls (SNK) test was utilized by utilizing letters with significant differences. Pearson correlation was used to determine the correlation between the chemical compounds measured and the antioxidant activity. Also, regression was applied to determine how altitude affects the tannin content. The level of *p* value < 0.05 was used to determine statistical significance.

## 3. Results and Discussion

### 3.1. TPC

**Figure 1:**
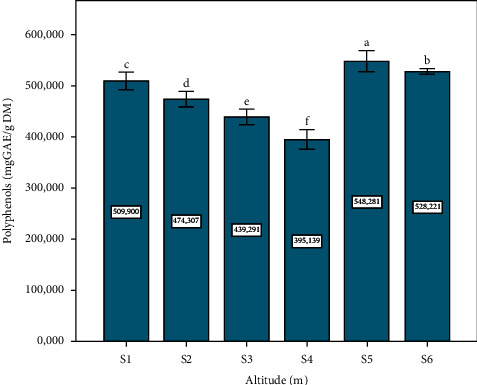
Total polyphenols content depending on altitude (m). Data means were calculated from three replications. The standard deviation of the mean is represented by the vertical bars. According to the SNK test, a significant difference is indicated when value do not share a common letter (*p* ≤ 0.05). S: site collection. The altitudes of sites collection are as follows: S1 = 1100 m, S2 = 1010 m, S3 = 800 m, S4 = 670 m, S5 = 500 m, and S6 = 390 m. CE: cyanidin equivalent, DM: dry matter.

According to results obtained (Figure 1), the highest polyphenol content was recorded in the plant leaves of site S5, followed by site six, where the results were 548.28 and 528.22 mg GAE/g DM, respectively, with a significant difference (*p* ≤ 0.05). Hadini et al. [38] and Gardeli et al. [30] reported the highest phenolic content of 541.92 mg GAE/g and 588 mg GAE/g, respectively, in *Pistacia lentiscus* leaves, and its results are consistent with those obtained. On the other hand, it was higher than those reported by Amel et al. (216.289 ± 20.62 mg GAE/g DM) [39]. The causes of these variations in the polyphenol content of leaf extracts may be due to, according to other studies, the influence of various factors such as altitudes (m) and the process of the technological extraction applied [7, 40]. In our study, we found a high content of polyphenols in the leaf extract at low altitudes (S5 = 500 m and S6 = 390 m), which can be explained by the reaction of plant materials to various types of stress caused by many factors that affected the number of phenolic compounds and improved metabolism of phenylpropanoids [41, 42].

### 3.2. TFC

**Figure 2:**
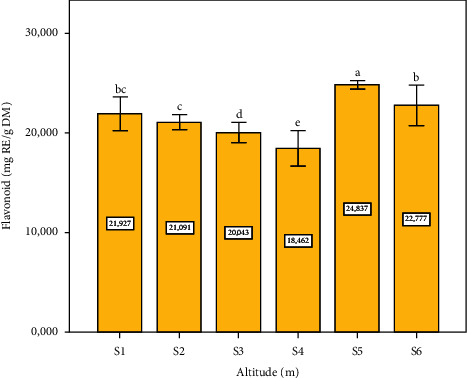
Flavonoid concentration depending on altitude (m). Data means were calculated from three replications. The standard deviation of the mean is represented by the vertical bars. According to the SNK test, a significant difference is indicated when values do not share a common letter (*p* ≤ 0.05). S: site collection. The altitudes of sites collection are as follows: S1 = 1100 m, S2 = 1010 m, S3 = 800 m, S4 = 670 m, S5 = 500 m, and S6 = 390 m. CE: cyanidin equivalent, DM: dry matter.

The obtained results from the quantification of flavonoid content in the *Pistacia lentiscus* L. leaves extracts showed that the higher content was observed in site five (Figure 2). Flavonoids belong to a substantial group of medicinally essential chemical compounds, regarded as a part of the process of synthesizing polyphenols and play role against oxidative stress situations, which justify our results obtained is very compatible with the variations in polyphenol concentration on the six sites' collections. The total flavonoid content of *P. lentiscus* leaves extract was higher than those reported by Atmani et al. [43] and Soumia et al. [44] (12.93 ± 1.69 mg/g and 8.21 ± 0.09 mg/g, respectively). On the other hand, our results are consistent with other research studies; for example, Hadini et al. [38], who obtained 22.33 mg RE/g DM. The flavonoids are intensively used in the food industry as flavoring agents and preservatives [45] because they improve the nutritional value of foods and their quality and also retard oxidative degradation [46], as skin protectors in the cosmetic industry [47], and as an anti-infective agent in agriculture practices [48]. These results justify the traditional use and application of the different organs of this medicinal plant by the local population in this area for the treatment of certain diseases.

### 3.3. CTs

**Figure 3:**
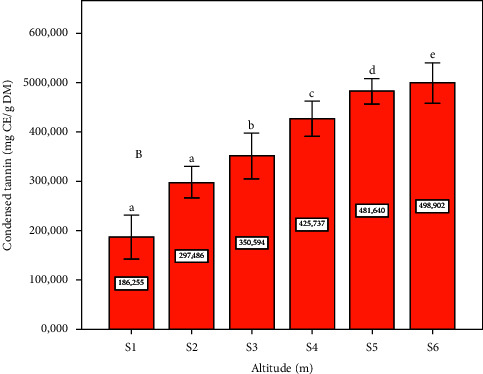
Condensed tannin contents depending on altitude (m). Data means were calculated from three replications. The standard deviation of the mean is represented by the vertical bars. According to the SNK test, a significant difference is indicated when value do not share a common letter (*p* ≤ 0.05). S: site collection. The altitudes of sites collection are as follows: S1 = 1100 m, S2 = 1010 m, S3 = 800 m, S4 = 670 m, S5 = 500 m, and S6 = 390 m. CE: cyanidin equivalent, DM: dry matter.

**Figure 4:**
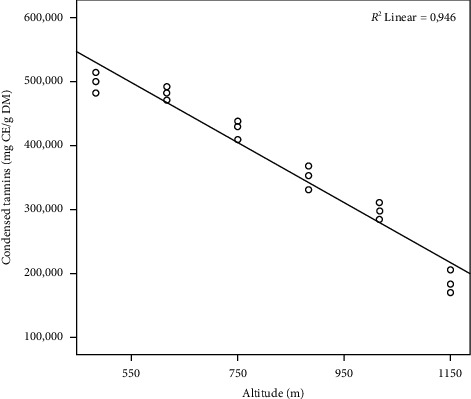
Linear regression curve between the altitude (m) and the concentrations of condensed tannins. CE: cyanidin equivalent, DM: dry matter.

The concentrations of CTs on the *Pistacia lentiscus* L. leaf at different altitudes (m) are presented in Figure 3. The comparison of the concentrations of CTs lentisk leaves extract showed that the most significant concentrations are marked at low altitudes (site six and site five) with a statistically significant difference (*p* ≤ 0.05) (Figure 3). In contrast, tannins are present in the leaves of lentisk with high concentration, especially at low altitudes (m). In comparison with our results, tannins in *P. lentiscus* L. leaves show high contents of 909.4 ± 42.61 mg CE/g DE, which was found by Atmani et al. [43]. Several research studies have shown that the different plant materials are rich and have a considerable variation in the tannin content [49]. Also, they have a beneficial effect on ruminant feed (they contribute to and facilitate the absorption of amino acids in the small intestine and protect them against the consequences of gastric juice). These results explain the use of this medicinal plant (leaves) in animals to treat a significant gastric problem [50]. In the current study, the concentrations of CT show a significant negative correlation with altitude (Table 1); when the altitude (m) is low, the tannin concentration content in the leaves will be high (Figure 4). Our results are in accordance with those of Hadini et al., who observed a negative correlation between tannin concentration and altitude in two different zones [38].

### 3.4. AscA Contents

**Figure 5:**
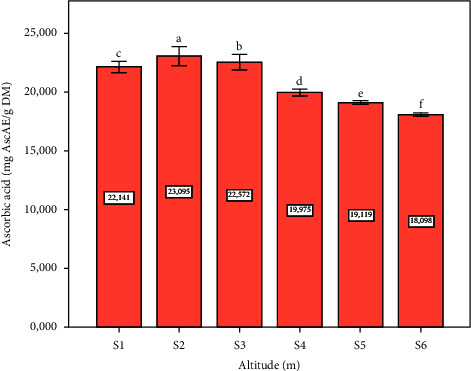
Ascorbic acid concentration depending on altitude (m). Data means were calculated from three replications. The standard deviation of the mean is represented by the vertical bars. According to the SNK test, a significant difference is indicated when values do not share a common letter (*p* ≤ 0.05). S: site collection. The altitudes of sites collection are as follows: S1 = 1100 m, S2 = 1010 m, S3 = 800 m, S4 = 670 m, S5 = 500 m, and S6 = 390 m. AscAE: ascorbic acid equivalent, DM: dry matter.

Figure 5 illustrates AscA concentrations measured at six sample sites. The highest concentration is observed at the site two, with a statistically significant difference (*p* ≤ 0.05). In plants, AscA comes mainly from the leaves [51]. It offers plant protection from oxidative damage as an antioxidant that can be dissolved in water, essential in plant cells [52]. On the other hand, AscA is a key player in plant photosynthesis, growth, and stress responses, with recent advances in understanding its biosynthesis paving the way for further exploration of its multifaceted roles [30, 53, 54]. Determination results of the AscA concentrations showed that this concentration is high in the leaves of *Pistacia lentiscus* L. It can be valued and utilized in different domains as a vitamin C.

### 3.5. Photosynthetic Pigment

**Figure 6:**
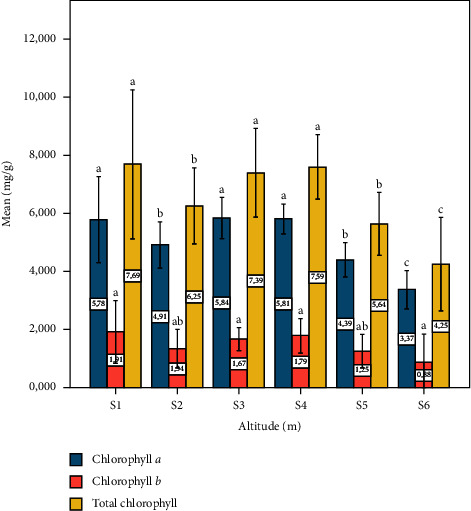
Photosynthetic pigment content depending on altitude (m). Data means were calculated from three replications. The standard deviation of the mean is represented by the vertical bars. According to the SNK test, a significant difference is indicated when values do not share a common letter (*p* ≤ 0.05). S: site collection. The altitudes of sites collection are as follows: S1 = 1100 m, S2 = 1010 m, S3 = 800 m, S4 = 670 m, S5 = 500 m, and S6 = 390 m.

The results of the determination of photosynthetic pigment contents in the leaf extracts of *Pistacia lentiscus* L. obtained at different levels of altitude (m) of both study sites are shown in Figure 6. The highest total chlorophyll, chlorophyll *a*, and chlorophyll *b* contents were observed in site one (1100 m); on the contrary, the minimum concentration is observed in site six (390 m). Generally, the chlorophyll contents are more important at high altitudes. These molecules can be found in several forms [55]; chlorophyll *a* and *b* present the most common. The concentrations of chlorophyll *a* and *b* in plants depend on the influence of many environmental factors related to humidity, temperature, and light exposure, which vary with altitudes [56]. Our results showed higher chlorophyll content than chlorophyll *b* at the six study sites. On the other hand, a minimum concentration of total chlorophyll is observed on site six. These results are consistent with those of Hadini et al. [38]. This could be seen as a protective mechanism in the case of plant stress [57, 58]. In addition, the high chlorophyll contents in the other sites may be due to the cycle of carbon minimizing or protecting the chloroplasts from oxidative damage.

### 3.6. In Vitro Antioxidant Assays

**Figure 7:**
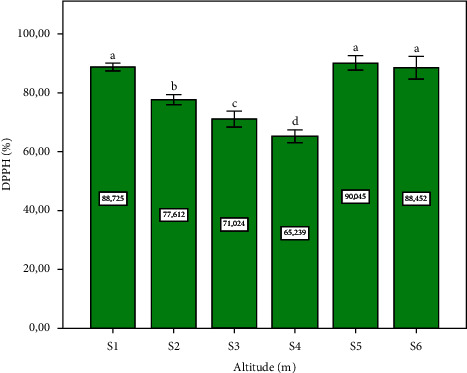
Leaves extract of *Pistacia lentiscus* L. on the 1,1-diphenyl-2-picrylhydrazyl (DPPH) activity depending on altitude (m). Data means were calculated from three replications. The standard deviation of the mean is represented by the vertical bars. According to the SNK test, a significant difference is indicated when values do not share a common letter (*p* ≤ 0.05). S: site collection. The altitudes of sites collection are as follows: S1 = 1100 m, S2 = 1010 m, S3 = 800 m, S4 = 670 m, S5 = 500 m, and S6 = 390 m. DPPH, 1,1diphenyl-2-picrylhydrazyl.

**Figure 8:**
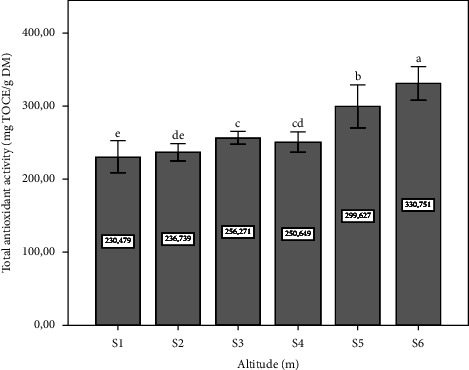
The total antioxidant activity depending on altitude (m). Data means were calculated from three replications. The standard deviation of the mean is represented by the vertical bars. According to the SNK test, a significant difference is indicated when values do not share a common letter (*p* ≤ 0.05). S: site collection. The altitudes of sites collection are as follows: S1 = 1100 m, S2 = 1010 m, S3 = 800 m, S4 = 670 m, S5 = 500 m, and S6 = 390 m. TOCE: *α*-tocopherol equivalent.

The results obtained are given in Figure 7. Our results show that a higher percentage of inhibition activity of DPPH was found in tree sites (S1, S5, and S6) with a statistically significant difference (*p* ≤ 0.05).  Figure 8 shows the results of the total antioxidant activity, expressed as mg TOCE/g DM. Site six (altitude: 390 m) showed the highest concentration, with a significant difference (*p* ≤ 0.05). Prior research aligns with our findings, demonstrating that *P. lentiscus* L. leaves collected from higher altitudes (1400 m and 720 m) exhibit enhanced antioxidant properties, with reported values of 87.31% and 88.55%, respectively. This observation is further corroborated by the current study's DPPH assay results from site one, which indicated an antioxidant activity of 88.72% [38]. Studies confirm the potent antioxidant properties of *Pistacia lentiscus* extracts, particularly from leaves than that of the other parts [59, 60]. This activity, comparable to strong antioxidants, neutralizes free radicals and protects cells from damage [61, 62]. The evaluation of the correlation between the antioxidant activity obtained and the phytochemical content, as given in Table 1, indicated that the DPPH correlated with the concentration of polyphenols. This result corresponds with many previous studies, confirming that the polyphenols are responsible for antiradical activity [63, 64]. Although there is a correlation between the total antioxidant activity and the flavonoid content (Table 1). Collectively, these provide compelling evidence for a positive correlation between altitude and the antioxidant potential of *P. lentiscus* L. leaves. Our results are in accordance with the literature search which indicated that the antioxidant activity in the *Pistacia lentiscus* leaves is related to secondary metabolite compounds characterized in our study [65].

## 4. Conclusion

In this work, we have opted for the study to measure the essential phytochemical constituents' content, their antioxidant capacities by DPPH activity, and the total antioxidant capacity from the leaf extracts from the eastern region of Morocco. Results of this study depicted that *Pistacia lentiscus* L. leaves extracts were potential sources of chemical compounds such as flavonoids, phenols, AscA, and tannins; they also had the greatest total antioxidant capacity assays and antioxidant activity in the DPPH. The present work proved that the different levels of altitude factors had no significant influence (*p* < 0.05) on the concentration of contents of the chemical compounds other than tannins. In addition, there was an essential significance of these second metabolites at low altitudes (m). Based on these results, the methanolic extract of *Pistacia lentiscus* could be greater source of essential phytochemicals that can be exploited and valorized in different domains. However, further research is needed to explore other molecular aspects of secondary metabolites by purifying the molecules of interest and to valorise the plant in the fields of agrifood and cosmetics.

## Figures and Tables

**Table 1:** Correlation results between the phytochemical analysis, antioxidant, and DPPH activity.

		Polyphenols	Flavonoids	Total antioxidant	DPPH	Tannins	Ascorbic acid
Polyphenols	Pearson correlation	1	0.941^2^	0.528^1^	0.979^2^	0.137	−0.374
	Sig. (2-tailed)		0.000	0.024	0.000	0.589	0.126

Flavonoids	Pearson correlation	0.941^2^	1	0.545^1^	0.901^2^	0.255	−0.406
	Sig. (2-tailed)	0.000		0.019	0.000	0.307	0.095

Total antioxidant	Pearson correlation	0.528^1^	0.545^1^	1	0.483^1^	0.838^2^	−0.873^2^
	Sig. (2-tailed)	0.024	0.019		0.042	0.000	0.000

DPPH	Pearson correlation	0.979^2^	0.901^2^	0.483^1^	1	0.047	−0.365
	Sig. (2-tailed)	0.000	0.000	0.042		0.853	0.136

Tannins	Pearson correlation	0.137	0.255	0.838^2^	0.047	1	−0.830^2^
	Sig. (2-tailed)	0.589	0.307	0.000	0.853		0.000

Ascorbic acid	Pearson correlation	−0.374	−0.406	−0.873^2^	−0.365	−0.830^2^	1
	Sig. (2-tailed)	0.126	0.095	0.000	0.136	0.000	

Abbreviation: DPPH, 1,1diphenyl-2-picrylhydrazyl.

^1^Correlation is significant at the 0.05 level (2-tailed).

^2^Correlation is significant at the 0.01 level (2-tailed).

## Data Availability

The data used to support the findings of this study are available from the corresponding author upon request.
